# Is implementation science a science? Not yet

**DOI:** 10.3389/fpubh.2024.1454268

**Published:** 2024-10-16

**Authors:** Dean L. Fixsen, Melissa K. Van Dyke, Karen A. Blase

**Affiliations:** Active Implementation Research Network, Inc., Chapel Hill, NC, United States

**Keywords:** science, implementation, independent variable, measurement, theory, fidelity

## Abstract

Getting the science right for implementation is critical for making the processes for improving outcomes more predictable and effective in global public health. Unfortunately, “implementation science” has become a catchphrase for ideas, assumptions, and findings concerning the science to service gap and how to close it. The purpose of this paper is to explore the dimensions of a “science of implementation” that meets the definitions of a science and is focused on implementation variables (i.e., purposeful processes to put innovations into effect so that intended benefits can be realized). A science of implementation is important for accomplishing the goals related to improving the health and well-being of populations around the world. Much of public health involves interaction-based interventions. In a typology of science, interaction-based interventions are created by specifying the nature of certain exchanges between and among individual people or groups. The complexity of developing interaction-based independent variables requires meeting benchmarks for fidelity to assure the presence and strength of implementation independent variables. The paper presents information related to the following tenets: (1) A science of implementation is based on if-then predictions. Science is cumulative. As predictions are made, tested, and elaborated, the facts accumulate to form the knowledge base for science and practice. (2) Implementation variables are interaction-based inventions and, therefore, must be created and established so the specific set of activities related to implementation can be studied. (3) A science of implementation is based on theory that organizes facts, leads to testable predictions, and is modified or discarded based on outcomes. (4) A science of interaction-based implementation depends on frequent measures of independent and dependent variables specific to implementation methods and outcomes. Two examples illustrate the implications for theory, research, and practice. The paper advocates a paradigm shift to a new mental model that values fidelity over tailoring, has one size fits all as a goal, and is concerned with the function of evidence rather than the form of evidence based on RCTs. Global health fundamentally requires scaling implementation capacity so that effective innovations can be used as intended and with good effect to achieve population benefits.

## Introduction

Since its beginnings in policy research and behavioral sciences in the 1960s (1–10) “implementation science” has become a catchphrase, a label for loosely related ideas, assumptions, and findings. There is nothing wrong with implementation science as a label, but it should not be confused with a science of implementation.

The National Institutes of Health (NIH) defines implementation science as the study of how to integrate evidence-based practices into routine health care and public health settings. The goal of implementation science is to improve population health outcomes. In contrast, a “science of implementation” is broader and open to learning about implementation in any domain.

In its current form, “implementation science” lacks organizing themes, agreed-upon language, and research focus. For example, Beidas et al. (11) summarized several “self-critical assessments” and added their own assessment of how recent decades of implementation science have not realized its goals of “achieving population health impact and social justice at scale.” Instead, decades of research has seen “the emergence of a new field with no common body of facts.” To counter “concerns that the field may be stagnating,” they advocated for less emphasis “on becoming a legitimate science” and encouraged the pursuit of simpler implementation strategies while acknowledging “the important role of multilevel context in implementation.” In contrast, a “science of implementation” is based on adhering to the basic tenets of science and acknowledges that implementation methods must account for the complexity of implementation challenges.

Science is based on testing predictions and accumulating facts so that phenomena of interest become more predictable and effective. A “science of implementation” is focused on factors related to changing practitioner, organization, and system behavior so that innovations can be used fully and effectively to reliably produce desired outcomes on a useful scale.

A science of implementation is important for accomplishing the goals for improving the health and well-being of populations around the world. Implementation is one of three factors that interact to produce socially important benefits for populations. The formula for success postulates (12–14):

Effective Innovations X Effective Implementation X Enabling Contexts = Socially Significant Benefits.

In the formula for success, an Effective Innovation is one that is defined and operationalized, and includes a way to assess fidelity of its use in practice; Effective Implementation refers to the interactive factors required to support the full and effective use of innovations in practice; and an Enabling Context is a system where the components are aligned so they support one another in coherent and purposeful ways so that improved system performance and improved outcomes can be achieved year after year. Socially Significant Benefits are outcomes that make a meaningful difference for the population of interest.

The formula is clear: if Effective Implementation is weak, socially significant benefits will be modest. The current “evidence-based movement” (15, 16) has focused on effective innovations and not effective implementation (17, 18) while giving worried attention to enabling contexts (19–21). The current extensive focus on “evidence-based interventions” has not led to substantial (purposeful, replicable, sustainable) improvements in public health. There are now many “evidence-based” innovations and too few socially significant benefits.

The purpose of this paper is to explore the dimensions of a “science of implementation” that make the processes for creating change more predictable and effective. In this paper, “implementation science” and “science of implementation” will be used without the quotes and are intended to mean two distinct things with (sadly) very little in common. The following sections explore the characteristics of a science of implementation and offer illustrative examples.

### Science of implementation

A science of implementation is based on if-then predictions. Science is cumulative. As predictions are made, tested, and elaborated, the facts accumulate to form the knowledge base for science and practice.

What qualifies as “science?” That is, how does science differ from other human activities: what discriminates astronomy from astrology, chemistry from alchemy, facts from beliefs (22, 23)? And, what discriminates a science of implementation from implementation science?

The philosophy of science is concerned with what science is and the logic for developing scientific knowledge. Fundamentally, science requires clearly stated predictions (if-then) and testable hypotheses (24–26). The predictions are falsifiable, and outcomes are replicable. For example, Galileo predicted (1604) that if any two or more objects of any size or weight are dropped from any height, then (without the interference of atmosphere) the objects will reach the ground at the same time. The prediction was supported (not falsified) as it was tested in Earth’s atmosphere. Newton developed a mathematical formula (1687) to calculate more precisely the predicted effects of gravity anywhere in the universe. Newton’s formula was used to determine the thrust to launch the astronauts from Earth and plot the complex four-day course to land them on the moon (1969). In 1971 (367 years after 1604) astronauts were able to meet the “without the interference of atmosphere” condition and tested Galileo’s prediction in the zero atmosphere conditions on the moon.^1^ Einstein (1905) expanded the theory of gravity and calculated its predicted effects on spacetime. This prediction was tested during the solar eclipse in 1919, the predicted position of the stars did appear to shift, and the theory of relativity gained major support.

Science is cumulative as exemplified in the discussion of gravity. Predictions generate facts, facts are organized in theories, and knowledge accumulates as facts reveal more of the fundamental truths and operations of general principles. Predictions tell us *if* something works (e.g., Galileo). Then scientists figure out *how* it works so it can be repeated (e.g., Newton). Finally, science may discover *why* something works (e.g., Einstein: all objects with mass bend and curve the fabric of the universe, called spacetime, and that curvature is felt as gravity).

Making predictions, testing predictions in experiments, and revising the knowledge base is not a common part of research under the current banner of implementation science. A search in Google Scholar for “implementation science” in the years 2005–2024 produced nearly 500,000 returns in June 2024. Prediction: no more than one published paper in any randomly selected set of 100 “implementation science” papers will include an explicit if-then statement regarding the purpose of the paper. Readers are encouraged to test each prediction in this paper as a way to hone the distinctions between a science of implementation and implementation science.

### Discoveries and inventions

A science of implementation is based on interaction-based inventions that describe when, where, with whom, and how specified interactions between people should occur to support the full and effective use of an innovation.

Implementation variables often are described as complex “reciprocal interactions” among multiple actors (27). “While the components of a system, namely the agents and their artifacts, are important, they are often secondary to the relationships between these components. In such systems, agents communicate and learn from each other and from their environment and adjust their behavior accordingly. However, there are many cross-cutting interconnections and influences” [(19), p. 5]. The assorted ideas that characterize implementation science likely result from attempts to deal with the inherent complexity of human behavior noted in this paragraph.

**Figure 1:**
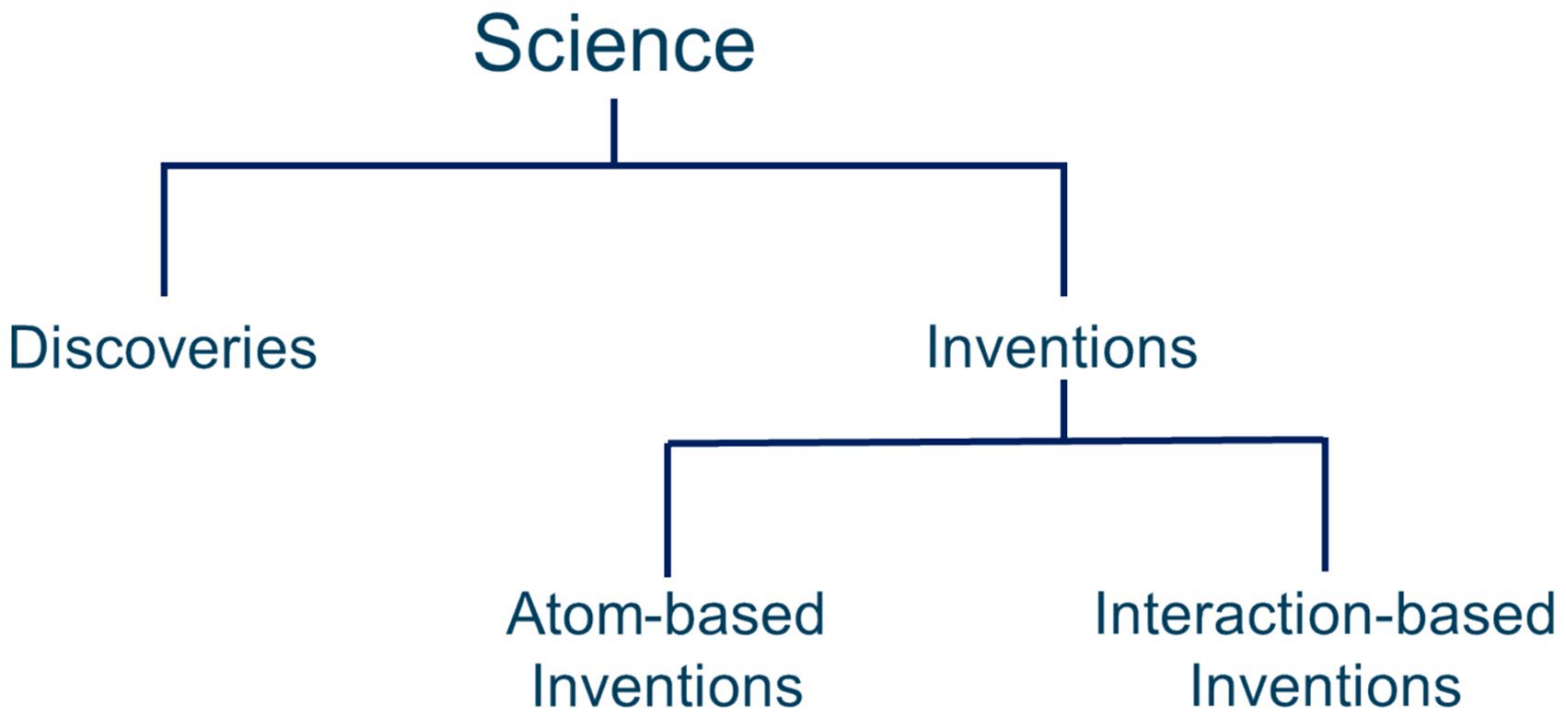
A typology of science, based on Wootton (28).

The source of this complexity can be better understood as the types of science are understood. Science is divided into two types as shown in Figure 1: discoveries and inventions (28). Previously unknown things that exist in nature can be discovered and science has generated facts and theories to describe and understand those discoveries. For example, geography and cartography and seismology evolved from the discovery of new lands and their shifting topographies. Chemistry evolved from the discovery of elements in the earth, water, and air. In these and other “natural sciences” such as biology, botany, physics, and astronomy the subject matter already exists and is available for study almost anywhere in the world.

Inventions are not “natural,” they are created. For example, light bulbs, telephone systems, integrated circuits, pain pills, highways, and office buildings are inventions. Computer science, engineering sciences, and so on evolved from the millions of inventions that have been created (29). In his book *The invention of science*, Wootton (28) documents the creation of science as it is understood today and notes that science itself is an invention, not a product of nature.

This distinction is recognized in law as well as in science. For example, a patent may *not* be issued for the discovery of a previously unknown phenomenon of nature. A patent can be issued only for an invention that applies a “law of nature” to a new and useful end.

In science, inventions are divided into two major subtypes as shown in Figure 1: atom-based inventions and interaction-based inventions [(30, 31), p. 22].

Atom-based inventions are created by using discoveries in new ways. For example, chemicals may be combined to produce a serum or a plastic object that is not found in nature but is based on natural (already discovered) elements. Or natural metals and crystals may be combined in new ways to form a transistor or silicon-based integrated circuit. After they are created, atom-based inventions are very stable (firmly established, enduring, resistant to change). As independent variables (if this) in tests of predicted outcomes (then that), atom-based inventions do not change from one use to the next or from one user to the next. Once they are created, they are available for study at the convenience of the scientist.

Gertner (29) described the success of Bell Labs, perhaps the most productive scientific enterprise of all time, averaging more than one patent per day for over 50 years. “If an idea begat a discovery, and if a discovery begat an invention, then an innovation defined the lengthy and wholesale transformation of an idea into a technological product (or process) meant for widespread practical use… An innovation could fail for technical reasons (if it proved unreliable) or for manufacturing reasons (if it proved difficult to reproduce consistently or cheaply).” Once they are created, atom-based innovations are relatively stable, predictable, and replicable.

Interaction-based inventions are created by specifying the nature of certain exchanges between and among individual people or groups (e.g., health workers and neighbors, implementation specialists and therapists, directors, and staff). The history of a piece of iron ore makes no difference in the makeup of an atom-based invention. However, in interaction-based inventions the history and ongoing life experiences of each individual or group will influence the quality of each example of an invention. Interaction-based inventions reflect the complexity of human behavior. After they are created, interaction-based inventions may not be stable as “recursive feedback constantly changes both agents, which in turn changes the other, again and over again” (32). As independent variables (if this) in tests of predicted outcomes (then that), the interaction-based invention may change from one use to the next and from one user to the next. Interactions are short-lived, and therefore interaction-based inventions are not available for study without careful preparation and monitoring by the scientist (33–35).

Compaoré et al. (36) studied the use of the WHO recommended intervention for Plasmodium falciparum malaria entitled Seasonal Malaria Chemoprevention (SMC). “Paired community distributors (CDs) dispense a full course of SMC drugs each month to targeted children using a door-to-door approach under the supervision of formal health workers. Each monthly drug distribution or SMC cycle lasts over 4 days, with an interval of exactly one month between two cycles. A complete course of SMC comprises a single treatment with SP and three daily doses of AQ; the CDs provide the first dose of AQ with SP to each 3- to 59-month-old child under directly observed treatment (DOT) [fidelity] in the absence of any contraindications.” Interaction-based innovations involve people working with people in planned ways where plans could go awry for many reasons. For example, Compaoré et al. (36) reported “difficulties due to insufficient training of community distributors, inadequate supply of inputs and insufficient financial resources for remuneration, advocacy and supervision, [and] the contextual constraints due to the rainy season.” None of these factors would influence the integrity of an atom-based innovation.

Understanding that interaction-based inventions are relatively unstable when compared to atom-based inventions is not an invitation to “tailoring” as commonly advocated in implementation science. Instead, it places the onus on implementation scientists to standardize interaction-based implementation methods to improve the stability and reliability of independent variables. In science, altering methods alters outcomes. Fidelity benchmarks set the minimum acceptable standard and exceptions are managed so that performance is sustained within an acceptable range so that predicted outcomes can be achieved more consistently. Meeting benchmarks for fidelity establishes a new normal distribution of competencies and outcomes and scientists can learn from the 15% top performers as they work to find ways to improve the 15% poor performers. In this way, fidelity provides a firm foundation for improvement using PDSAC improvement cycles (37) or Total Quality Management methods (38).

Assuring the integrity of the independent variable is not a common part of research under the banner implementation science. Prediction: no more than one published paper in any randomly selected set of 100 “implementation science” papers in the years 2005–2024 will clearly identify an implementation independent variable and make an if-then prediction of its effects.

### Implementation

Implementation variables are interaction-based inventions and, therefore, must be created and established so that the specific set of activities related to implementation can be studied.

Implementation is the purposeful process of putting something into effect. Implementation is:

“a specified set of activities designed to put into practice an activity or program of known dimensions. According to this definition, implementation processes are purposeful and are described in sufficient detail such that independent observers can detect the presence and strength of the ‘specific set of activities’ related to implementation. In addition, the activity or program being implemented is described in sufficient detail so that independent observers can detect its presence and strength. When thinking about implementation, the observer must be aware of two sets of activities (intervention-level activity and implementation-level activity) and two sets of outcomes (intervention outcomes and implementation outcomes)” [(39), p. 5].

As reflected in this definition, implementation is universal and applies to any situation where there is an attempt to “put into practice an activity or program [an innovation] of known dimensions.” For any innovation, implementation methods (independent variables) can be studied to see the effects they may have on “putting the innovation into practice.” For a science of implementation, the focus is not on innovations but rather on factors that support the full and effective use of innovations in practice. Like statistics, implementation is universal and exists independently of any particular area of application. The support methods, not the innovations, are the focus of a science of implementation. Thus, a science of implementation can benefit from studies done in any field.

#### Interaction-based independent variables in a science of implementation

Implementation factors that support the full and effective use of innovations are interaction-based inventions. They do not exist in nature waiting to be discovered; they are created and specify the interactions between and among people.

Interaction-based independent variables add considerable difficulty to the development of a science of implementation. The implementation independent variable must be established by a research team at the time and place an experiment is to be conducted, and in sufficient quantity and with sufficient quality to satisfy the requirements of an experimental design. This, in itself, is an implementation challenge. In a science of implementation every dependent variable at one level is an independent variable at the next level [(31), p. 13ff].

“A research group must be sufficiently skilled in using implementation practice (the independent variable) so that practitioners will use an innovation with fidelity (the dependent variable). At the next level, the practitioners’ use of an innovation with fidelity (the independent variable) is assessed in terms of benefits to recipients (the dependent variable). [Thus], the fidelity of practitioners’ use of an innovation is both a dependent variable and an independent variable.”

This means a research team must be knowledgeable and skilled in the use of implementation best practices so that interaction-based independent variables can be established at sufficient strength to be studied. In effect, the research group functions as an implementation team that uses current implementation best practices to establish variables that can be studied to create new implementation knowledge. This is similar to atom-based software development where it is common practice to develop software using the software being developed, such as using and improving barely functional MS Word 0.0 *en route* to developing MS Word 1.0 for the intended end users (40, 41).

Implementation applies to itself as the first users of new knowledge are the scientists themselves. Armed with new knowledge, scientists can establish and test more robust and complete interaction-based independent variables to test riskier predictions. A science of implementation depends on implementation practice.

**Figure 2:**
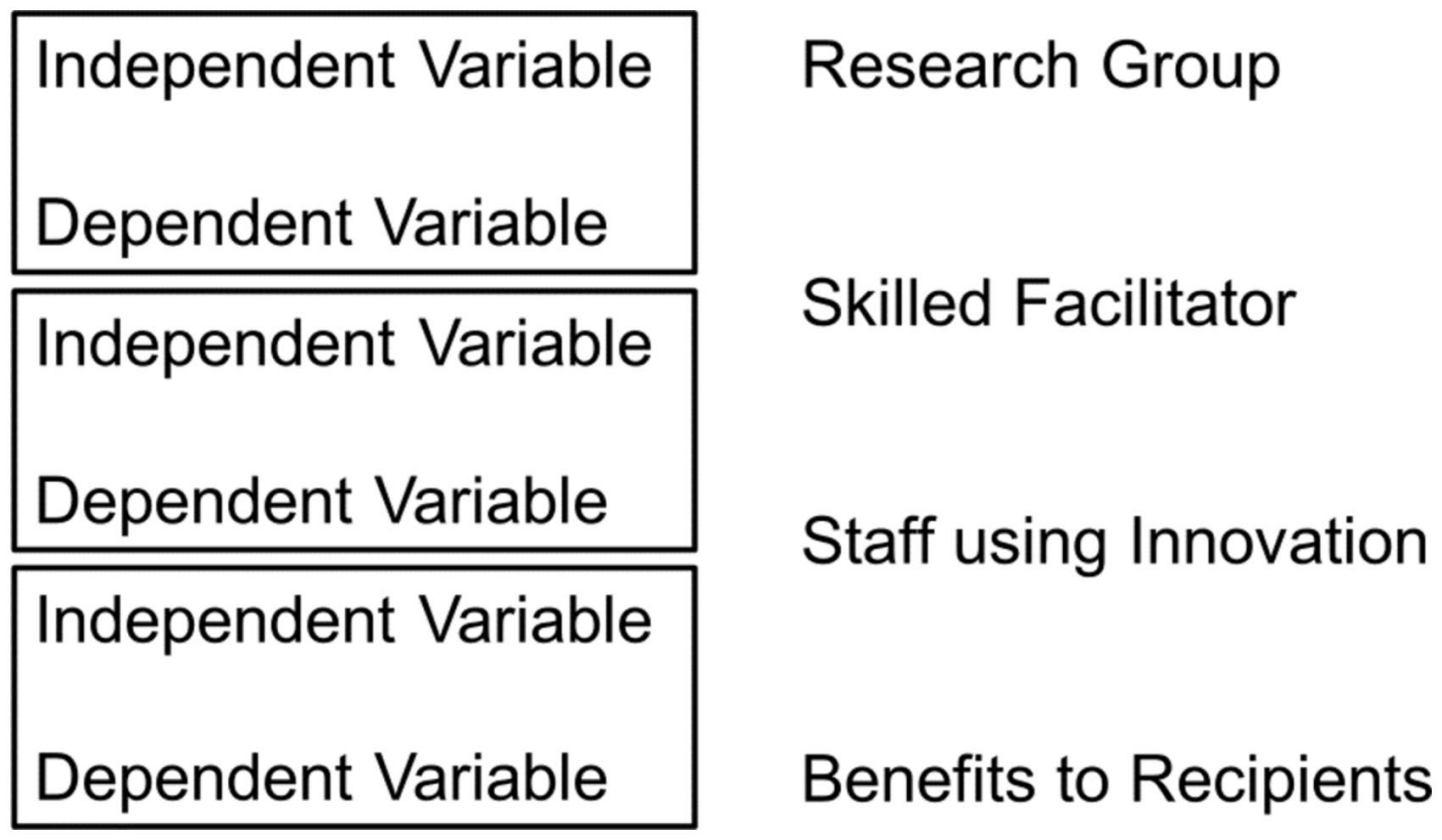
Implementation interaction-based variables where dependent variables also function as independent variables.

The logic for research on implementation variables is shown in Figure 2. In this example “facilitation” is the implementation independent variable. Skilled facilitators must be established by the research group so that facilitation is available for study. The intended outcome of facilitation (the implementation independent variable) is increased use of a specific innovation (the implementation dependent variable). Use of the innovation as intended (the innovation independent variable) is expected to result in benefits to recipients (the innovation dependent variable). Given that implementation is universal, the same implementation methods (e.g., training, coaching, fidelity to develop competencies) can be used to establish the independent variable at each level of use (research group creating skilled facilitators; skilled facilitators creating staff skills to use the innovation). Thus, skilled facilitators in this example (simultaneously) are the outcome of the research group and the input for staff using the innovation.

With respect to fidelity of the independent variable at each level, An et al. (42) recommend establishing pre-set benchmarks that must be met before a study is conducted. For example, a large-scale study (over 4,000 households) employed specially trained community health workers (CHWs) who delivered individual and combined water, sanitation, handwashing (WSH) and child nutrition interventions to the homes and families of pregnant women in rural villages in Bangladesh. In this study, the fidelity of delivery of the innovation was assessed proactively from the beginning. After three months, fidelity (in the 30–60% range) was found to be well below the pre-set benchmark of 80% for each independent variable. The research group made adjustments to improve the delivery of CHW services, and the fidelity scores improved (in the 86 to 93% range). At the end of the study the multiple benefits of the complex WSH intervention were substantial (43, 44). The research group produced skilled CHWs who produced high fidelity examples of the combined intervention and, therefore, the independent variable was available for study and was found to be effective for producing improvements in water quality, handwashing, sanitation, and nutrition.

Assessing fidelity and using 80% as a benchmark for fidelity helps to assure the intervention “is there.” Given that implementation variables are interaction-based inventions, implementation scientist teams must create sufficient implementation practitioner expertise so that implementation variables can be established at sufficient strength for study.

Implementation science does not insist on fidelity assessments to assure the presence and strength of interaction-based independent variables. Methods for establishing independent variables are glossed over if they are included at all (e.g., “the practitioners were trained to use the innovation”). Thus, learning “what works” for putting something into effect has not been cumulative for practice or science. Prediction: no more than one published paper in any randomly selected set of 100 “implementation science” papers in the years 2005–2024 will clearly identify the implementation independent variable and report the fidelity of its use during the study.

### Theory

A science of implementation is based on theory that organizes facts, leads to testable predictions, and is modified or discarded based on outcomes.

In science, theory is a source of predictions (if-then) that lead to observations to confirm or disconfirm theory-based predictions. Theory is improved or discarded based on the outcomes of theory-based predictions. In this way, science is cumulative with today’s knowledge built on the foundations of past predictions that were tested and led to improved theory (45).

Five criteria that define a theory were outlined by Carpiano and Daley (46).

1. Logic: The major concepts and relations should be logically coherent. Terms must be clearly defined so that they can be understood by those examining the theory.
2. Causality: The goal of theoretically based research is to identify the systematic components of a set of factors that produce change in the phenomena being studied. Causal drivers and a sense of causal process should be clearly identified.
3. Falsification: The major propositions should be empirically falsifiable. All useful theories suggest ways in which they may be subjected to empirical assessment.
4. Scope: Although it can change over time, the scope of the theory should be clear and relatively broad. It must be focused on generic processes and not unique characteristics of any specific situation or case.
5. Productivity: The theory should promote non-obvious implications (“risky predictions”) and produce a relatively large number of predictions per assumption.
  Popper (25) (p. 39) adds another criterion:
6. Prohibition: Every good scientific theory forbids certain things to happen. The more a theory forbids, the better it is.

The explicit predictions made in this paper are examples that meet Popper’s prohibition criterion.

With respect to Carpiano and Daley’s criterion regarding scope, the Improved Clinical Effectiveness through Behavioral Research Group (47) distinguishes among grand theories, mid-range theories, and micro-theories. These are summarized in Table 1.

**Table 1:** Levels of theory as defined by the improved clinical effectiveness through behavioral research group (47).

Scope	Definition	Purpose
Grand or Macro Theory	A grand or macro theory is a very broad theory that encompasses a wide range of phenomena	A grand theory is a general construction about the nature and goals of a discipline
Mid-Range Theory	A mid-range theory is more limited in scope, less abstract, addresses specific phenomena, and reflects practice. It encompasses a limited number of concepts and a limited aspect of the real world.	Mid-range theory is designed to guide empirical inquiry. Mid-range theories are made up of relatively concrete concepts that are operationally defined and relatively concrete propositions that can be empirically tested.
Micro Theory	A micro-practice, or situation-specific theory (sometimes referred to as prescriptive theory) has the narrowest range of interest.	Micro theory focuses on specific phenomena that reflect clinical practice and are limited to specific populations or to a particular field of practice.

Implementation frameworks can qualify as theory. Carpiano and Daley (46) define a conceptual framework as a specific set of variables and the relations among them that are presumed to account for a set of phenomena. There are multiple implementation frameworks (48–50) that could be assessed as fitting the definition of mid-range theories and could serve as a source of predictions (if-then) in a science of implementation.

To use theory to advance science, Gelman and Shalizi (45) suggest “we build a … model out of available parts and drive it as far as it can take us, and then a little farther. When the model breaks down, we dissect it and figure out what went wrong.” “The goal is to learn about general laws, as expressed in the probability that one model or another is correct.”

Given that implementation practice and science are focused on outcomes (*if* we do this, *then* that occurs *so that* people benefit), consequential validity is an essential test of any theory-based prediction. Consequential validity favors external validity over internal validity (51, 52). Galea (53), working in a health context, described consequential validity:

A consequentialist approach is centrally concerned with maximizing desired outcomes …. A consequentialist epidemiology inducts new trainees not around canonical learning but rather around our goals. Our purpose would be defined around health optimization and disease reduction, with our methods as tools, convenient only insofar as they help us get there.

By thinking of “our methods as tools, convenient only insofar as they help us get there,” the consequential validity question is “so what?” and the prediction/conclusion statement is “so that” (a phrase used throughout this paper). Once an implementation variable is postulated, it is incumbent on the researcher (the if-then predicter) to provide data that demonstrate how knowing that information “helps us get there” (i.e., behavior change so that outcomes are achieved).

A theory often is mentioned and rarely tested, improved, or discarded in implementation science. Prediction: no more than one published paper in any randomly selected set of 100 “implementation science” papers in the years 2005–2024 will clearly state how the study was designed to test a prediction based on an explicit theory of implementation.

### Measurement

A science of interaction-based implementation depends on frequent measures of independent and dependent variables specific to implementation methods and outcomes.

Given the relative instability of interaction-based innovations, accurate measurement is paramount. A scientist (and the audience) must be assured that the implementation factor (if this: the independent variable) is present and at sufficient strength so that the results (then that: the dependent variable) reasonably can be attributed to the implementation factor (54–58). For interaction-based innovations, the independent variable must be measured repeatedly throughout an experiment with the same accuracy and care as the dependent variable.

For example, McIntosh et al. (59) examined fidelity of the use of Positive Behavior Interventions and Supports (PBIS). PBIS is an evidence-based multifaceted whole school intervention to reduce student discipline problems and improve academic outcomes. PBIS is conducted in the context of complex local and state education systems. The PBIS innovation involves all teachers and all students in each school along with parents, community members, and education system staff. The study included 5,331 schools located in 1420 school districts in 37 states. Each school that met fidelity criteria for the first time between 2005 and 2009 was included. The fidelity data for each school was tracked for the following Years 1–5. The researchers found four distinct patterns of the use of PBIS over five years. There were two patterns of sustained implementation (sustainers and slow starters) and two patterns of abandonment (late abandoners and rapid abandoners). In Year 1, 3,904 of the schools continued to use PBIS as intended (i.e., met fidelity criteria). In Year 3, 2,735 schools met fidelity criteria and in Year 5, 2,239 schools met fidelity criteria. Given the patterns of use shown in the data, different schools did or did not meet fidelity criteria each year, the exception being the subgroup of sustainers (29% of the 5,331 schools). Thus, for individual schools, PBIS “was there” in some years and “not there” in others. With multiple interaction-based variables in play, knowledge of the fidelity of the use of PBIS (the independent variable) is essential for interpreting any outcome data, and assessing fidelity at one point in time was insufficient.

The relative instability of interaction-based implementation methods makes fidelity an essential implementation variable in every study. Fidelity answers the question: Is “it” there? When “it” is there a scientist can ask: Does “it” matter? Assessing fidelity is important for any independent variable (54), and especially important for interaction-based implementation variables. For example, are the “methods to assure executive leadership understanding and support” being used as intended (with fidelity)? Are the “methods to support practitioner competency development” being used as intended (with fidelity)? Are the “methods to create effective implementation teams” being used as intended (with fidelity)? If “it” is there, then the outcomes of “it” can be studied. Without an assessment of fidelity, it is difficult to know the extent to which all, some, or none of any intended method has been delivered. “Without fidelity measures, treatment becomes a mysterious black box: We do not know precisely what the intervention is, how to implement it, and what quality of it has been delivered. The black-box approach represents pre-scientific clinical care” [(60), p. 881].

Fortunately, fidelity is being recognized as a critical implementation measure across a number of fields using interaction-based inventions (61–71). Nevertheless, fidelity is not a requirement and is not commonly assessed in implementation science. Prediction: no more than one published paper in any randomly selected set of 100 “implementation science” papers in the years 2005–2024 will clearly state how the independent variable in the study was created/established and include an assessment of the presence and strength of the independent variable during the study.

## Two examples

As outlined in this paper, the science of implementation will advance as evidence is generated to test predictions based on theory, interaction-based implementation variables are measured to assure their presence and strength, and theory is adjusted or discarded based on new evidence.

Evidence is “facts, information, etc. that give reasons for believing that something is true or present”.^2^ Currently, implementation science judges evidence by the type of research design (72). This focus on form (evidence is the product of a research design) is seen to “contribute to the … implementation gap” (73). In a science of implementation evidence is judged by its function (evidence that a variable depends on and varies with another). External validity and consequential validity are primary considerations and “our methods [are] tools, convenient only insofar as they help us get there.”

To establish a science of implementation, we propose the application of the following nine criteria to guide the development of research studies, as well as the analysis of the quality of the research design. In a science of implementation, evidence (the function of the evidence) can be judged using the following nine criteria.

1. Is the implementation independent variable clearly identified?
2. Is the implementation independent variable explicitly related to a theory?
3. Is the implementation independent variable explicitly related to implementation (i.e., the purposeful process of putting something into effect)?
4. Are the methods clearly stated for purposefully establishing the implementation independent variable (e.g., creating staff and management readiness, developing staff competencies, changing organization routines)?
5. Are the methods for establishing the implementation independent variable assessed (e.g., pre-post training tests of knowledge and skill, coaching done by a skilled coach)?
6. Is the implementation independent variable measured to detect its presence and strength (e.g., facilitation was done as intended)?
7. Is the implementation independent variable assessed frequently during the study (e.g., a minimum of three times, no more than three months apart)?
8. Does the implementation dependent variable focus on changes in behavior (e.g., practitioners, organizations, or systems)?
9. Are the results used to confirm, modify, or discard a theory?

These questions are based on the forgoing discussion of science, interaction-based variables, theory, and measurement. To illustrate the application of these questions, two reviews are provided. Research conducted by Acosta et al. (74) is an excellent example that meets all nine criteria. Research conducted by Seers et al. (75) was augmented by post-hoc analyses (76, 77) and is presented as the second example. In the comments below, the answers to the nine questions primarily are excerpts (without quote marks) or paraphrased from the cited documents. Thus, any first-person pronouns (e.g., I, we) in the answers below refer to Acosta et al. or Seers et al. The studies cited below were not conducted with the questions in mind. A science of implementation will benefit from using the criteria as a guide for planning future implementation research.

Prediction: no more than one published paper in any randomly selected set of 100 “implementation science” papers in the years 2005–2024 will meet five or more of the nine “(function of) evidence” criteria outlined for a science of implementation.

1. Is the implementation independent variable clearly identified?
  - Yes. Assets Getting To Outcomes (AGTO) is an implementation support intervention that consists of a manual of text and tools, face-to-face training, and onsite technical assistance, focused on activities shown to be associated with obtaining positive results across any prevention program.
2. Is the implementation independent variable explicitly related to a theory?
  - Yes. The current study is the first to evaluate the Getting to Outcomes Framework (GTO) on both individual capacity and program performance in a way flexible enough to account for the ‘complex, interacting, multi-level, and transient states of constructs in the real world’. The Getting to Outcomes Framework is a mid-level theory of implementation.
  - The AGTO model operationalizes the Consolidated Framework for Implementation Research (CFIR) to ensure that all the major domains that influence implementation are considered. Consistent with CFIR, the study attempts to evaluate AGTO in terms of its impact on three of the CFIR domains: inner setting, individual characteristics, and implementation process, while using measures of the outer setting as covariates. CFIR is a mid-level theory of implementation.
3. Is the implementation independent variable explicitly related to implementation (i.e., the purposeful process of putting something into effect)?
  - Yes. As assessed in the survey, prevention capacity was defined as efficacy and behaviors of practitioners and relates to CFIR’s individual characteristics domain. To assess AGTO’s potential impact on each program’s implementation process, we first documented the degree to which each program engaged in the AGTO intervention through the AGTO participation index, which is the sum of six true/false items added to the mid and post survey.
4. Are the methods clearly stated for purposefully establishing the implementation independent variable (e.g., creating staff and management readiness, developing staff competencies, changing organization routines)?
  - Yes. The AGTO intervention includes three types of assistance which are adapted to fit the needs and priorities of the individuals involved, as well as the inner and outer setting: a manual of text and tools, face-to-face training, and onsite technical assistance (TA). These three types of assistance aim to improve the implementation process for each program. Two full-time, Maine-based staff, one with a master’s and one with a bachelor’s degree, provided AGTO tools, training, and TA to the intervention coalitions and programs during the two-year intervention period. The tools are in the Search Institute-published manual, Getting To Outcomes with Developmental Assets: Ten steps to measuring success in youth programs and communities, which all intervention participants received. The training was delivered separately to each coalition over a full day after baseline, and covered the AGTO model, tools in the manual, and an introduction to the TA process. Based on TA literature, the AGTO-based TA involves three structured steps, including an initial diagnosis of program functioning, development of a logic model, and development of a plan for how the TA and program staff were to make improvements, carried out during and in between biweekly TA visits. TA staff provided consultation and feedback to practitioners on conducting AGTO tasks as applied to their program.
5. Are the methods for establishing the implementation independent variable assessed (e.g., pre-post training tests of knowledge and skill, coaching done by a skilled coach)?
  - Yes. To assess AGTO’s potential impact on each program’s implementation process, we first documented the degree to which each program engaged in the AGTO intervention through the AGTO participation index, which is the sum of six true/false items added to the mid and post coalition survey. Based on the Hall et al. model of categorizing the degree to which individuals ‘use’ an innovation, these items assess key markers of use including participation in training, reading the materials, planning, discussing the model with colleagues, securing resources, and receiving TA. Exposure to AGTO was also documented by TA providers recording hours of TA they delivered to each program, by AGTO step. The Participation Index and hours of TA have been shown to be related to prevention capacity and performance in a previous study of GTO.
6. Is the implementation independent variable measured to detect its presence and strength (e.g., facilitation was done as intended)?
  - Yes, see above. A structured interview was used to assess the impact of AGTO on the implementation process, administered on the same timeline as the survey.
7. Is the implementation independent variable assessed frequently during the study (e.g., a minimum of three times, no more than three months apart)?
  - Yes, see above, twice for AGTO and incrementally as GTO segments were taught and used.
  - The survey assessed prevention capacity defined as efficacy and behaviors of practitioners and relates to CFIR’s individual characteristics domain. The Asset Behaviors scale includes five items assessing whether individuals are motivating both adults and youth to become asset-builders; incorporating asset building into existing youth programs; influencing community leaders to implement asset-aligned policies.
8. Does the implementation dependent variable focus on changes in behavior (e.g., practitioners, organizations, or systems)?
  - Yes. Capacity of coalition members and performance of their programs were compared between the randomly selected groups (n = 6 in each) across the baseline, one-, and two-year timepoints.
  - A structured interview was used to assess the impact of AGTO on the implementation process, administered on the same timeline as the coalition survey. Prevention practitioners performance of tasks associated with high-quality prevention targeted by AGTO were captured through the interview. Whole programs are rated, not individuals, because programs operate as a unit.
  - The amount of change between the intervention and control groups did not significantly differ across the three time points for assets efficacy, GTO behaviors, or for any of the other prevention capacity scales in tests of condition by time interaction.
9. Are the results used to confirm, modify, or discard a theory?
  - Yes. Using the implementation research typology outlined in CFIR, this study evaluated the AGTO intervention’s impact on the capacity of individual prevention practitioners (i.e., CFIR’s individual characteristics) and the performance of whole programs (CFIR’s implementation process), while accounting for several factors in CFIR’s inner setting domain.
  - The methods used in the current study also have implications for broader implementation research using the CFIR. The study used measures to assess the quality of the implementation process as well as the relevant capacity of practitioners (i.e., individuals involved) that could be adapted to many other interventions and exist already for interventions in the areas of substance abuse prevention, positive youth development, prevention of teen pregnancy and sexually transmitted infections, and homelessness prevention. These measures were used in a randomized controlled trial and our analytic approach addressed certain challenges of conducting research on real-world implementation (e.g., the turnover of membership at participating coalitions, differential response rates between intervention and control groups). More innovative methods and more acceptance of nontraditional research methods will be needed to capture the complex, multi-level and transient status of constructs in the real-world as the study of implementation evolves.

The following nine questions and answers pertain to the Seers et al. studies.

1. Is the implementation independent variable clearly identified?
  - Yes. In the context of the PARIHS framework, facilitation refers to the process of enabling (making easier) the implementation of evidence into practice. Thus, facilitation is achieved by an individual carrying out a specific role (a facilitator), which aims to help others. This indicates that facilitators are individuals with the appropriate roles, skills, and knowledge to help individuals, teams, and organizations apply evidence into practice…. To fulfill the potential demands of the role, facilitators likely will need a wide repertoire of skills and attributes.
2. Is the implementation independent variable explicitly related to a theory?
  - Yes. The PARIHS framework (78) is a mid-level theory of implementation that predicts a key role for facilitation in producing implementation outcomes. A test of this theory-based prediction was conducted by a PARIHS group of researchers (75–77).
3. Is the implementation independent variable explicitly related to implementation (i.e., the purposeful process of putting something into effect)?
  - Yes. In theory, if staff in an organization use facilitation as a process, then the organization will be more likely to translate research evidence into practice.
4. Are the methods clearly stated for purposefully establishing the implementation independent variable (e.g., creating staff and management readiness, developing staff competencies, changing organization routines)?
  - Yes. To teach the wide repertoire of skills and attributes needed by facilitators, the research team prepared two different facilitator development programs, each of which involved an initial residential program, followed by virtual support (monthly telephone group supervision and email communication) for the internal facilitators (IFs) in implementing the urinary incontinence (UI) recommendations.
5. Are the methods for establishing the implementation independent variable assessed (e.g., pre-post training tests of knowledge and skill, coaching done by a skilled coach)?
  - No. The methods section identifies two implementation independent variables, (1) training (initial residential program) and (2) coaching (followed by virtual support), and one implementation dependent variable (prepare the internal facilitators). The effects of training and coaching were not measured during the experiment or in post-hoc analyses. Staff behavior also was not measured, a critical link between the potential impact of facilitators on staff behavior and patient outcomes.
6. Is the implementation independent variable measured to detect its presence and strength (e.g., facilitation was done as intended)?
  - No.
7. Is the implementation independent variable assessed frequently during the study (e.g., a minimum of three times, no more than three months apart)?
  - No. Fidelity (facilitation as provided by the internal facilitators) was not measured during the study but was assessed retrospectively with record reviews and interviews (76).
8. Does the implementation dependent variable focus on changes in behavior (e.g., practitioners, organizations, or systems)?
  - No. The primary outcome was the documented percentage compliance with continence recommendations produced by the fourth International Consultation on Incontinence. Percentage compliance is calculated for each resident, so outcomes are measured at the resident level.
9. Are the results used to confirm, modify, or discard a theory?
  - No. Facilitation as defined by PARIHS was not present and, therefore, the predicted influences of facilitation and the PARIHS theory were not tested in this study.

## Discussion

Is implementation science a science? Not yet. This paper summarizes the dimensions of a science of implementation and provides two examples of how those dimensions relate to the evidence on which a science of implementation can be based.

- A science of implementation is based on if-then predictions. Science is cumulative. As predictions are made, tested, and elaborated, the facts accumulate to form the knowledge base for science and practice.
- Implementation variables are interaction-based inventions and, therefore, must be created and established so the specific set of activities related to implementation can be studied.
- A science of implementation is based on theory that organizes facts, leads to testable predictions, and is modified or discarded based on outcomes.
- A science of interaction-based implementation depends on frequent measures of independent and dependent variables specific to implementation methods and outcomes.

The loosely related ideas, assumptions, and findings currently under the label implementation science fall far short of meeting the qualifications to be considered science, and little of the literature relates to purposeful implementation processes created to put something into effect. Despite the massive investment in the “evidence-based movement” in the past three decades, the science to service gap remains and the contributions of implementation science to society have been modest at best. Six explicit predictions are made in this paper (“no more than one published paper in any randomly selected set of 100 “implementation science” papers in the years 2005–2024…”). The predictions likely will hold up if they are tested, a clear indication that implementation science is not a science of implementation. Following the tenets of science, if this-then *not* that, it may be time to give up that paradigm and try something new.

The lack of socially significant outcomes was noted early in the current evidence-based movement. Perl (18) was concerned about the “addiction” to creating new evidence-based programs and Kessler and Glasgow (52) called for a 10-year moratorium on efficacy-focused randomized controlled trials (RCTs) in health and health services research. These authors encouraged an investment in a science of implementation. Calling for a “revolution,” Kruk et al. (79) (p. 1196) note that “Changing health needs, growing public expectations, and ambitious new health goals are raising the bar for health systems to produce better health outcomes and greater social value. But staying on current trajectory will not suffice to meet these demands.”

The revolution begins with changes in how a science of implementation is understood. Specifically, the characteristics of a science as outlined in this paper challenge deeply held beliefs about implementation: what it is and how to develop new knowledge about it.

First, a science of implementation argues against “tailoring” as advocated in implementation science (80, 81). A science of implementation is based on understanding that it is necessary for scientists to assure the fidelity of the independent variable to counter the inherent instability of interaction-based inventions. An assessment of fidelity (is it there?) is a requirement. Encouraging users to alter methods may increase their acceptability to users but not their benefits to recipients. In science, changing methods changes outcomes.

Second, the goal of science is to establish general truths about how things work. In this sense, it is a quest to develop implementation methods where “one size fits all.” Any plan for scaling for population impact will consist of many one-size-fits-all processes so that “it” can be done with fidelity many thousands of times with improvement methods built into the process. “Build a … model out of available parts and drive it as far as it can take us, and then a little farther. When the model breaks down, we dissect it and figure out what went wrong” [(45), p. 32]. As the science and the processes of one size fits all are put into practice, evidence is generated and double loop learning can occur. In this way the pool of effective methods expands to incorporate effective responses to what previously was unanticipated. The expanded methods then can benefit a greater proportion of the variations encountered in communities, service settings, and organizations. The new, more robust “one” then can bene-“fit” a greater proportion of “all.”

Third, if-then predictions can be tested in many ways to demonstrate functional relationships between implementation independent and dependent variables (if this is done, then that happens). The Acosta et al. (74) example demonstrates how each implementation variable (participation in training, reading the materials, planning, discussing the model with colleagues, securing resources) can be assessed for fidelity (is “it” there?) in the context of doing multifaceted work in complex environments over longer periods of time. The first question is, can it be done at all, even once? Given the counterfactuals for interaction-based variables that must be invented (it has never been done, not even once), pre-post experiments may be sufficient to assess strong variables and learn how to create them on demand. Thinking of randomized control trials (“RCTs”) as the gold standard emphasizes form over function. While RCTs may be appropriate later on as the science matures, initially they get in the way of creating powerful independent variables and developing a science of implementation.

These implications mean the “mental model” (82, 83) for implementation research and practice needs to change in pursuit of a science of implementation. A mental model is an intuitive perception about acts and their consequences, tacit assumptions of how the world works. How do we think about problems and potential solutions? As Ashby (84) said, “The fault cannot be in the part responsible for the repair.” Exhortations to “think outside the box” are invitations to change mental models. “Deep change in mental models, or double loop learning, arises when evidence not only alters our decisions within the context of existing frames, but also feeds back to alter our mental models. As our mental models change, we change the structure of our systems, creating different decision rules and new strategies” [(83), p. 509]. Tailoring, one size cannot fit all, and the value of RCTs are accepted truths in implementation science and are impediments to establishing a science of implementation.

Getting the science right for implementation is critical for global public health. Global health fundamentally requires scaling implementation capacity (i.e., purposeful processes to put something in place) so that effective innovations can be used as intended and with good effect for whole populations. As predicted in the formula for success, scaling effective implementation is critical for scaling effective innovations to achieve population benefits.

Atom-based innovations are scaled by developing manufacturing capacity. For example, Apple corporation has 615 production facilities (implementation capacity) that produce about 240 million iPhones (the innovation) per year. Apple designs the products, specifies the manufacturing processes, and requires 1,200 quality assurance staff in each contracted production facility to achieve 80% yield rates for products that pass final quality control standards (fidelity).^3^

Interaction-based innovations are scaled by developing implementation teams (implementation capacity). Scaling to achieve public health outcomes begins by developing a scalable unit (85). A scalable unit is an administrative unit that includes the practitioners who deliver the effective innovation; the effective implementation team that supports the selection, competency development, and fidelity assessment of practitioners; and administrators to assure an enabling context of funding, licensing, and so on. As noted in the description of the formula for success in the introduction, any one part of a scalable unit without the others will not produce and sustain local or (eventually) population benefits. Initial testing of the scalable unit can be done at a single site if that site represents key system components and relationships among components likely to be encountered in the system at full scale (85–87).

The methods to establish a scalable unit are detailed by Titler et al. (87) in their description of the Iowa Model of Research-Based Practice. “The practice is first implemented with a small group of patients, and an evaluation is carried out. The EBP is then refined based on evaluation data, and the change is implemented with additional patient populations for which it is appropriate. Patient/family, staff, and fiscal outcomes are monitored. Organizational and administrative supports are important factors for success in using evidence in care delivery.” Thus, the elements essential to effective innovation, effective implementation, and enabling context factors that reliably produce socially significant outcomes are worked out before attempting scaling to achieve population benefits. Of course, yesterday’s solutions provide the platform for tomorrow’s problems, so the improvement processes never end until population goals are reached and sustained.

Scaling is possible only when scalable units are sustained. If already established units are dropping out or losing their effectiveness as fast as new units can be created, then scaling and socially significant benefits plateau at that point. Metaphorically, the public health container is draining as fast as it is being filled. Thus, testing of the scalable unit is essential. For example, Fixsen, Blase, Timbers, and Wolf (88) found when the scalable unit only focused on developing practitioners to deliver the effective innovation, 17% of the residential treatment units sustained for six or more years. When the focus shifted to developing whole organizations as the scalable unit so that the implementation team and administration also were included, 84% of the residential treatment programs sustained for six years or more. Testing the scalable unit and getting it right made a big difference in sustainability of effective implementation and innovation practices.

With a scalable unit tested and ready, the next task is to develop implementation teams at the district level whose purpose is to develop and support scalable units at the local level. Then, regional implementation teams must be developed to establish more district implementation teams and continually support them. And, finally, a national implementation team must be developed to establish and support a growing number of regional implementation teams. Establishing teams can be done simultaneously (or nearly so) in a top-down and bottom-up sequence so that the linked teams can be created expeditiously. The good news is that a science of implementation is universal, so the work of the various implementation teams is similar although with different goals (12, 89). The same concepts for scaling can be found in descriptions of holarchies (90) and fractals (32, 91) where units with similar functions are repeated at various points of scale. Common concepts, common language, and common measures promote clear communication and coordinated effort among the linked implementation teams [(31); Chapter 15].

Developing implementation capacity locally, regionally, and nationally was essential in the successful efforts to eradicate smallpox (accomplished in 1979). As described by Foege (92), thousands of local surveillance teams and containment teams (the effective innovation) were supported by hundreds of regional implementation teams (effective implementation) that were developed with the support of national and global governments and global health organizations (enabling contexts). In India there were 29 states and 386 districts subdivided into “blocks” (roughly 100,000 population in a block; a scalable unit for surveillance). Competency development for many thousand health workers had to be done well and be repeatable in several hundred training sessions provided at each level in each state. In India, training was provided for staff at each level: state, district, and block. In one state, preparations for the first search required over 60 training sessions at the regional level simply to get down to the district level, and an additional 930 training sessions at the district and block levels. This process for developing implementation capacity was repeated in the other 28 states so that effective surveillance teams and containment teams could reach the entire population of India (600 million). Implementation capacity was established, and smallpox was eradicated.

Developing implementation capacity locally, regionally, and nationally also is essential in the successful efforts to implement the National Health Policy in Ethiopia (beginning in 1998). The National Health Policy gives strong emphasis to fulfilling the needs of less privileged rural communities, which constitute about 83% of the total population of 90 million (93). Like the smallpox surveillance teams and containment teams for a “block” of 100,000 population, Ethiopia recruited and developed two HEWs (health education workers) for each “health post” serving 3,000–5,000 population. HEWs are recruited based on nationally agreed criteria that include residence in the village, capacity to speak local language, graduation from 10th grade, and willingness to remain in the village and serve rural and distressed communities. Selection is done by a committee comprising members nominated by the local community and representatives from the woreda (district) health office, the woreda capacity-building office, and the woreda education office. All selected HEWs go through a year-long training, which includes both theoretical training in training institutions and practical training in health centers. By 2014, over 30,000 HEWs had been deployed in health posts nationally.

Wang et al. (93) described linked implementation (“supervisory”) teams consisting of members from different disciplines at the federal, regional, and woreda levels in Ethiopia. The implementation teams are involved in all aspects of program management, including planning, implementation, and monitoring and evaluation. Members of each team are trained in skills needed for supportive supervision (facilitation, interpersonal communication, problem solving, and analytical skills); oriented to various tools and methods (such as peer review and performance assessment tools); and provided with opportunities to frequently upgrade their technical skills. The implementation team members are trained in a specially designed curriculum. At each level (federal, regional, and woreda), the implementation team prepares its own annual plan, checklists, and detailed schedule for each supervisory visit. The work of implementation teams cascades from regional level down to woreda, health center, and health posts. Implementation teams also actively engage regional and woreda councils for issues that go beyond the health sector itself (93).

As illustrated in these examples, realizing socially significant benefits requires effective innovations supported by effective implementation in enabling contexts. Each factor needs to be purposeful, functional, and improvable so that benefits to whole populations are good to start with and improve with experience. Without a science of implementation, global health and well-being will remain an aspiration and not an achievement.

A science of implementation is within our reach. If experiments focus on implementation variables and if independent and dependent implementation variables are measured accurately and repeatedly to determine their function, then a science of implementation can be developed, and populations can benefit.

## Data Availability

The original contributions presented in the study are included in the article/supplementary material, further inquiries can be directed to the corresponding author.

## Footnotes

^1^ https://www.youtube.com/watch?v=ZVfhztmK9zI

^2^ https://dictionary.cambridge.org

^3^ https://9to5mac.com/2023/01/17/iphone-quality-standards
